# Arthroscopic debridement of the osteoarthritic knee combined with hyaluronic acid (Orthovisc^®^) treatment: A case series and review of the literature

**DOI:** 10.1186/1749-799X-3-43

**Published:** 2008-09-17

**Authors:** Xinning Li, Agam Shah, Patricia Franklin, Renee Merolli, Jill Bradley, Brian Busconi

**Affiliations:** 1 University of Massachusetts Medical Center, Department of Orthopaedic Surgery, Division of Sports Medicine, 55 Lake Avenue North, Worcester, MA 01655, USA

## Abstract

**Objective:**

An evaluation of safety and efficacy of high molecular weight hyaluronan (HA) delivered at the time of arthroscopic debridement of the osteoarthritic knee.

**Methods:**

Thirty consecutive patients who met inclusion and exclusion criteria underwent arthroscopic debridement by a single surgeon and concomitant delivery of 6 ml/90 mg HA (Orthovisc^®^). These patients were evaluated preoperatively, at 6 weeks, 3 and 6 months post-operatively. Evaluations consisted of WOMAC pain score, SF-36 Physical Component Summary (PCS) score and complications.

**Results:**

No complications occurred during this study. Pre-op average WOMAC pain score was 6.8 +/- 3.5 (n = 30) with a reduction to 3.4 +/- 3.1 at 6 weeks (n = 27). Final average WOMAC pain score improved to 3.2 +/- 3.8 at six months (n = 23). No patients had deterioration of the WOMAC pain score. Mean pre-operative SF-36 PCS score was 39.0 +/- 10.4 with SF-36 PCS score of the bottom 25th percentile at 29.9 (n = 30). Post procedure and HA delivery, mean PCS score at 6 weeks improved to 43.7 +/- 8.0 with the bottom 25th percentile at 37.5 (n = 27). At 6 months, mean PCS score was 48.0 +/- 9.8 with the bottom 25th percentile improved to 45.8 (n = 23).

**Conclusion:**

The results show that concomitant delivery of high molecular weight hyaluronan (Orthovisc^® ^– 6 ml/90 mg) is safe when given at the time of arthroscopic debridement of the osteoarthritic knee. By delivering HA (Orthovisc^®^) at the time of the arthroscopic debridement, there may be a decreased risk of joint infection and/or injection site pain. Furthermore, the combination of both procedures show efficacy in reducing WOMAC pain scores and improving SF-36 PCS scores over a six month period.

## Introduction

Osteoarthritis (OA) is the most prevalent musculoskeletal disorder and the leading cause of disability in adults over the age 45 years [1-3]. OA of the knee is extremely common, with some studies showing that OA of the knee occurs in at least 30% of people after the age of 50 years, and in 80% of people older than 75 years [4,5]. Of the patients with OA, over 80% will have limitation of movement and greater than 25% can not perform their activities of daily living [3]. Traditionally, conservative treatment for knee OA has been symptomatic and includes basic analgesics, i.e. acetaminophen, non-steroidal anti-inflammatory drugs, intra-articular injection of glucocorticoids, activity modification, weight loss with exercise, and physical therapy. Recently, Anakinra, an IL-1 receptor antagonist (IL-1RA) has shown effectiveness in treating OA in a multi-center randomized clinical trial [6,7]. Furthermore, insulin as a slow releasing formulation may also have potential benefits in the treatment of OA [8]. Surgical treatments for patient refractory to conservative management include arthroscopic debridement plus joint lavage or unicompartmental/total joint arthroplasty.

Arthroscopy for knee OA has remained a topic of controversy amongst clinicians. Multiple clinical trials discuss the efficacy of arthroscopic debridement and/or lavage in the treatment of knee OA [2,9-21]. The procedure serves to remove the products of cartilage wear, mechanical irritations, inflammatory cells and molecules from the joint; therefore counter the onset of painful inflammatory phases of OA. Despite numerous studies, debate over the role, indications, and long term efficacy of arthroscopic debridement in the treatment of knee OA still exists [11,12,15,16,22]. Many investigators have sought for solutions to improve outcomes after arthroscopic debridement of knee OA, which may involve but not limited to better patient selection, improved operative technique, and/or well defined indications [11].

Recently, hyaluronan (HA) injection has contributed as a treatment modality for knee OA. HA is a type of glycosaminoglycan that naturally exists in the joint space and contributes to both the viscosity and lubrication in the normal synovial fluid. It has been studied as a substance capable of restoring the normal properties of synovial fluid and cartilage thus reducing pain and stiffness in the knee of patients with OA [23,24]. Several exogenous preparations of HA exist and are derived from avian or bacterial sources for the expected use of intra-articular injection. Named viscosupplementation, one proposed mechanism of action of HA injection is supplementing the viscous properties of altered pathologic synovial fluid [24,25]. HA may also have a protective effect on the chondrocytes and may include additional anti-inflammatory effects: inhibition of phagocytosis [26,27], prostaglandin synthesis [28], removal of oxygen-free radicals [29] and suppression of inflammatory cytokine activity [30]. Exogenous injection of HA may also stimulate *in vivo *synthesis of HA [24]. A recent animal study showed HA was able to inhibit IL-1beta induced chondrocyte apoptosis in a dose dependant manner [30].

Standard dosage of HA (Orthovisc^®^) is 2 ml (15 mg of HA/ml) injection per week for three consecutive weeks, which may be repeated in 4–6 months with the same protocol for OA of the knee. Several studies have documented the efficacy and safety of the use of HA supplementation (2 ml/30 mg/injection) in the OA patient [31-33], however, there are no studies to date that describe the concomitant use of 6 ml/90 mg (Orthovisc^®^) HA at the time of the arthroscopic procedure. The primary goal of our study was to determine the safety and documenting any complications after injecting 6 ml/90 mg of Orthovisc^® ^(3× the normal dose) into the knee. Our secondary goal was to evaluate preliminary efficacy (6 months follow-up) of combined concomitant HA delivery and arthroscopic debridement for OA of the knee utilizing health related questionnaires (WOMAC and SF-36).

## Methods and materials

This investigation was approved by and performed in accordance with the guidelines of the institutional review board at our hospital. Thirty consecutive patients undergoing knee arthroscopy between the ages of 21–65 years with knee osteoarthritis who met the inclusion and exclusion criteria were recruited for this study. This patient population consisted of nineteen males and eleven females with an average age of 46 years. All patients carried a preliminary diagnosis of osteoarthritis (via MRI and plain radiograph) and were candidates for primary arthroscopic treatment of a unilateral knee secondary to a meniscal pathology, loose bodies, or osteochondral defect. Failed conservative treatment modalities of at least 6 months included, but were not limited to activity modification, weight loss, physical therapy, and oral medication. Patients who were candidates for unicompartmental or total knee arthroplasty were excluded from this study. All patients presented with pain greater than or equal to 3/10 as measured by the Visual Analog Scale (VAS). The diagnosis of osteoarthritis was further confirmed at the time of arthroscopy with all included patients having at least a Grade II or III lesion per the International Cartilage Repair Society (ICRS) in one of the compartments of the knee. Any patients found to have rheumatoid arthritis, avascular necrosis, or any other of the inflammatory arthritis was excluded from this study. Patients were also excluded form the study if any of the following criteria was found to be pre-existing: Allergy to poultry or avian derived products, previous surgical interventions to the index knee, osteochondritis dessicans lesion greater than 7 mm in depth, Body Mass Index greater than 35, history of steroid injection to the effected knee within the past three months, history of hyaluronic acid injection within the past year, or moderate/greater symptomatic contra-lateral knee involvement.

All patients were enrolled after a complete history and physical. An MRI and plain radiographs including AP weight bearing, lateral, and merchant views of the involved knee were obtained. All of the patients included in our study had no radiographic evidence of severe degenerative joint disease as indicated by the presence of osteophytes, subchondral sclerosis and/or cysts. Furthermore, all of our study patients had normal knee alignment, which falls between 3 to 8 degrees of valgus with no flexion contractures per physical examination. Patients were asked to complete VAS, Western Ontario and McMaster Universities Osteoarthritis Index (WOMAC), and Short Form-36 Physical Component Summary questionnaires (SF-36 PCS.)

**Figure 1:**
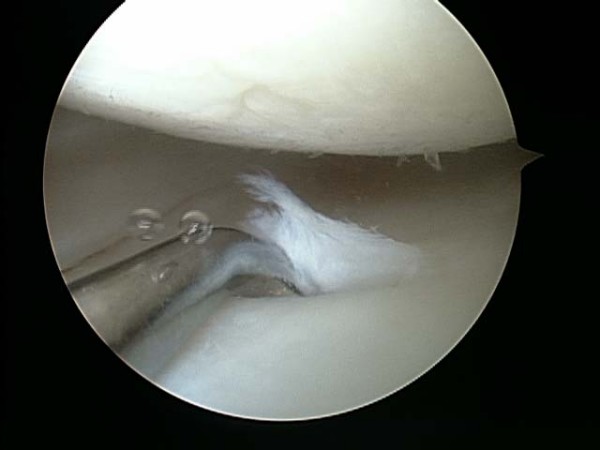
**Arthroscopic view of the medial femoral tibia joint.** A grade I (ICRS) lesion was noted on the medial femoral condyle and a grade III lesion with cartilage flap was seen on the medial tibia.

**Figure 2:**
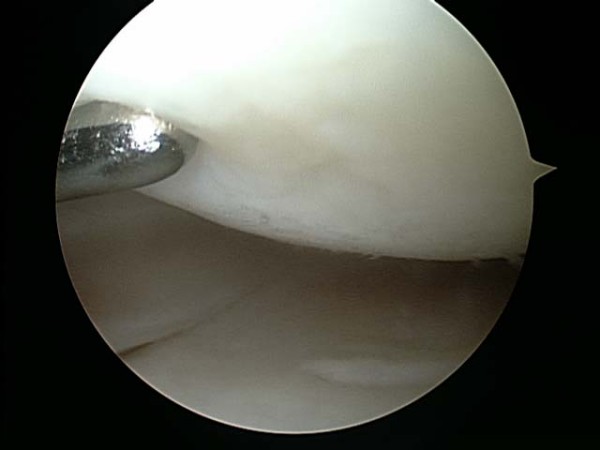
Post arthroscopic debridement of the chondral lesions on the medial femoral condyle and the medial tibia.

**Figure 3:**
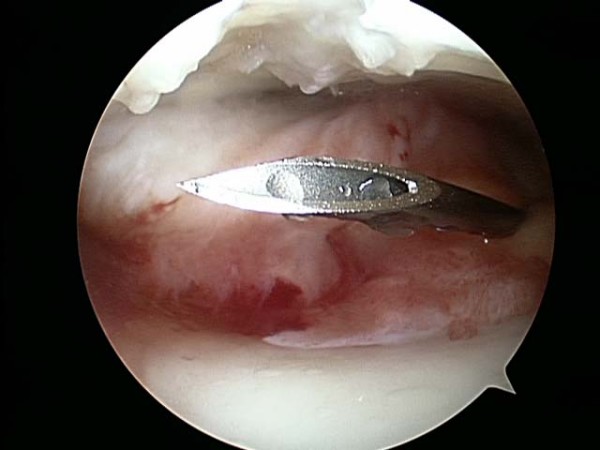
An 18 gauge needle is inserted into the patellofemoral joint under direct arthroscopic visualization.

**Figure 4:**
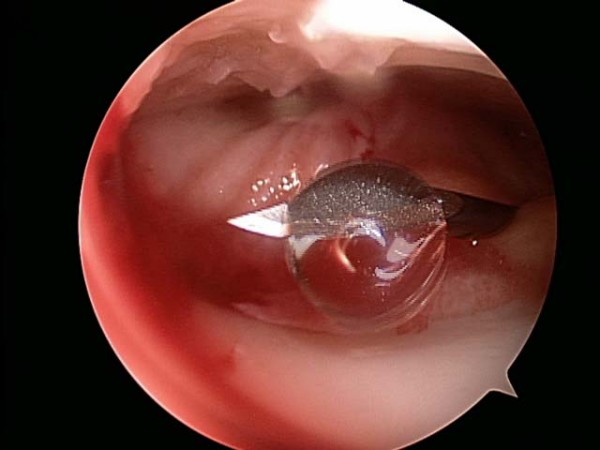
Hyaluronic Acid (6 ml/90 mg of Orthovisc^®^) is injected into the knee joint via an 18 gauge needle and under direct arthroscopic visualization.

Between May and July of 2006, a single surgeon (BB) performed all thirty knee arthroscopies. Standard medial and lateral parapatellar portals were established to perform the procedure. The patellofemoral joint, medial femoral tibial joint, and lateral femoral tibial joint were systematically evaluated. All compartments were graded using the ICRS grading scale: Grade I was assigned to cartilage that appeared nearly normal and had superficial lesions only, Grade II was assigned to abnormal appearing cartilage lesions that extended less than 50% of the depth of the cartilage, Grade III was assigned to cartilage lesions that extended greater than 50% of the depth of the cartilage or evidence of cartilage blistering or lesions down to the calcified layer, and Grade IV was assigned to lesions that extended to the subchondral bone plate. Chondroplasties were performed with a shaver if appropriate (Figure 1 and 2). Meniscal pathology was also noted and appropriately debrided to a stable state. The knee was thoroughly irrigated, all loose bodies were removed, and the knee joint was appropriately drained. Using the arthroscope for direct visualization (Figure 3), 6 ml/90 mg of high molecular weight hyaluronic acid (Orthovisc^®^, sodium hyaluronate; Anika Therapeutics, Inc, Woburn, MA) was delivered into the intra-articular space via an 18 gauge needle under direct visualization after the arthroscopic procedure (Figure 4). All portals were subsequently closed with interrupted nylon sutures before the injection of HA with the exception of the arthroscope portal. Standard dosage of HA (Orthovisc^®^) is 2 ml/30 mg (15 mg of HA/ml) per injection per week for three consecutive weeks. Thus injecting 6 ml/90 mg of HA (Orthovisc^®^) was three times the normal dosage. After injection, the arthroscope portal was closed with nylon sutures and sterile dressings were applied.

Post-operatively, patients were allowed to weight bear as tolerated with the use of crutches if necessary and were given a two week prescription for narcotic analgesics (Vicodin 5/500 mg) for pain control. Patients were asked to ingest one enteric coated aspirin per day for two weeks for DVT prophylaxis. The subjects were scheduled to have appointments at one week, six weeks, three months and six months post operatively. Any complications were documented at each visit. A physical therapy (PT) directed rehab protocol was implemented at one week as per the surgeon's standard of care. Each patient received three treatment of PT per week with no restrictions. All patients were asked to complete SF-36 PCS and WOMAC questionnaires at the pre op, six week, three month, and six month visits.

## Results

All thirty arthroscopy procedures were performed without any intra-operative complication. No complications were documented within the six-month study period to include, but not limited to infection, bleeding, nerve damage, deep venous thrombosis, pulmonary embolus, allergic reaction, and HA delivery site pain.

ICRS grading showed that the patellofemoral joint had the greatest amount of involvement with an average grade of 3.1 +/- 0.5. The medial compartment revealed an average ICRS grade of 2.8 +/- 0.8 and the lateral compartment revealed an average grade of 1.8 +/- 0.8. Twenty-six patients had meniscal tears that required debridement. All knees had chondroplasty of at least one compartment, which was performed with a shaver. Two of the thirty knees were found to have loose bodies greater than five millimeters in diameter.

Of the thirty patients receiving the index procedure, three were lost to follow-up at six weeks (n = 27) and an additional four patients were lost to follow-up at six months (n = 23). Thus, data was collected for twenty-seven patients at the six week and three month post-op visit and for twenty-three patients at the six month visit (77% at final follow up appointment).

**Figure 5:**
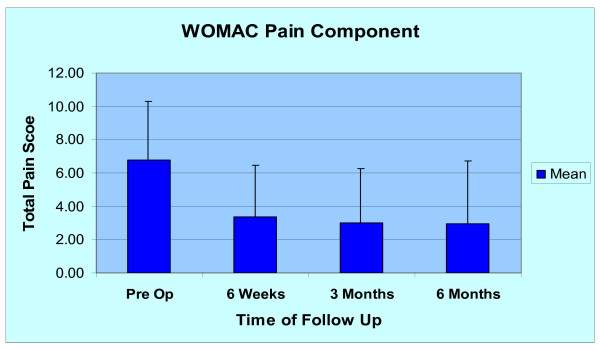
Mean WOMAC Pain Score of all patients at pre op, 6 weeks, 3 months, and 6 months.

The average pre-operative WOMAC pain score within the study group was 6.8 +/- 3.5 (n = 30). At the six-week post-operative visit, the average WOMAC pain score improved to 3.4 +/- 3.1 (n = 27). At the final six-month visit (n = 23), the average WOMAC pain score improved to 3.2 +/- 3.8 (Figure 5). Ninety-six percent (26/27) of patients had improvement in WOMAC pain scoring and one patient had no improvement at the 3 month follow up time. No patients had deterioration of post-operative WOMAC pain scores at the 6 months follow up appointment. The patients were further stratified into those having improvement greater than 15% with outcomes showing 87% of patients with sustained improvements at the final follow up (6 months).

**Figure 6:**
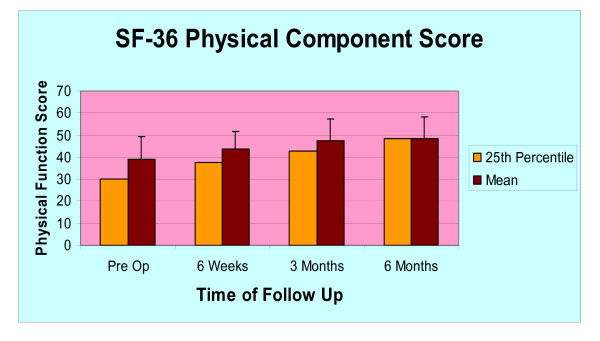
**Mean SF-36 Physical Component Summary (PCS) Score at pre op, 6 weeks, 3 months, and 6 months.** The red bar represents the mean scores of all patients in our study and the yellow bar represents the bottom 25th percentile of patients.

The average pre-operative SF-36 Physical Component Summary (PCS) score within the study group was 39.0 +/- 10.4 (n = 30). At the six-week post-operative visit, the average SF-36 PCS score improved to 43.7 +/- 8.0 (n = 27). At the final six-month visit, the average SF-36 PCS score improved to 48.0 +/- 9.8 (n = 23). The data was also stratified to calculate changes in the bottom 25^th ^percent of patients. The average pre-operative SF-36 PCS score within the lowest quartile was 29.9. At the six-week post-operative visit, the average SF-36 PCS score of the lowest quartile improved to 37.5. At the final six-month visit, the average SF-36 PCS score of the lowest quartile improved to 45.8 (Figure 6). Eighty-seven percent (20/23) of patients had improvement in the SF-36 PCS scores with three patients having had some deterioration of SF-36 PCS scores at the 6 month time point.

## Discussion

Traditional and labeled use of Hyaluronic Acid (HA) Orthovisc^® ^for treatment of pain due to OA of the knee involves a series of 2 ml (30 mg of HA: 15 mg/ml) injections weekly in the clinic over a span of three weeks. Some studies have documented the complication rate of this injection to be as high as 27% [34], with the most complication being injection site pain. Other complications of significance are transient local reactions in the injected joint, which are typically benign, short lived, without sequelae, and similar to those observed with any intra-articular treatment [35]. These adverse reactions have been found to occur at a rate of 2–4% [31].

In this study using 6 ml/90 mg of Orthovisc^®^, no complications were noted at the 6 months follow up time period. The presence of minimal joint swelling was observed in several patients at the earlier follow up appointments which can be attributed to the arthroscopic procedure; however, none of these patients were symptomatic and all resolved by the 6 months appointment. No patients had evidence of infection, transient reactions, deep venous thrombosis, or other neurovascular insult. Delivery site pain and reaction may have occurred but could remain disguised by post surgical pain or narcotic use (Vicodin). Because of these influences, the ability of our study to detect delivery site pain or other related symptoms were somewhat limited. Overall, we found that 6 ml/90 mg of Orthovisc^® ^can be given safely at the time of arthroscopy without any adverse complications. However, this statement can not be generalized to all HA supplements as Orthovisc^® ^was the only HA used in our study. Ulucay et al. showed no difference in the complications between three different HA formulations (Orthovisc, Adant, and Synvisc) after arthroscopic knee debridement for OA [36].

The dosing effect of giving 6 ml/90 mg of Orthovisc^® ^when compared to three separate injections raises concern. Several meta-analysis on investigating the safety and efficacy of HA injections show that the standard dosing is safe and effective [25,31,32]. Our pilot study results showed at least equivalent efficacy and safety at the six-month mark. We did not observe any adverse events at the 6 months time period with the increased dosage of 6 ml/90 mg of Orthovisc^®^. Bellamy et al. [37] in a meta-analysis found that WOMAC pain scores peaked between 5 and 13 weeks and tended to deteriorate towards baseline over 52 weeks. Brandt et al. [32] in a randomized control trial of 226 patients found that HA injections improved WOMAC pain scores for the duration of the study of 30 weeks. Neustadt et al. [33] in a randomized control trial of 272 patients followed over a period of 28 weeks showed that a WOMAC pain scores improved by greater than 40% in patients receiving HA when compared to those receiving placebo. Our study showed consistently improved WOMAC pain scores after 24 weeks with a single delivery of 6 ml/90 mg of Orthovisc^® ^at the time of the arthroscopic debridement with over 79% of our patients having an improvement of greater than 40%. However the final follow up in our study was at the six month mark, so we can not conclude that the improvement in WOMAC pain score would be sustained for greater than one year.

Dervin et al. [2] performed a study examining the outcomes of arthroscopic debridement alone on arthritic knees. They found 44% of the patients in the study sustained a significant decrease in WOMAC pain scores over two years, where improvement was considered greater than 15%. Their study population composed of 126 patients with knee OA that is refractory to conservative management and with significant medial compartment disease (57% had ICRS Grade III or IV lesions) and meniscal tear (63%) which is similar to the patient population of our study. Compared to this historical control, our study showed that WOMAC pain scores from the combined procedure was significantly improved in 87% of our patients and SF-36 PCS scores improved in 89% of patients at the 6 months follow up. Furthermore, the patients in the bottom 25th percentile of the SF-36 PCS in our study improved from 29.9 (pre-op) to 45.8 at the 6 months follow up. However, the length of follow up (6 months) may have skewed our results.

In another study by Mathies et al. [38] HA injection was performed after arthroscopy for meniscal tears. He found that patients had less pain at rest and during exercise when compared to a control group. Patients in the HA group were also noted to have less joint swelling and better Lysholm scores. Hempfling el al. [39] in a recent study compared arthroscopic knee debridement and lavage for knee OA versus the same procedure with immediate injection of 10 ml of HA post arthroscopy. A total of 80 patients were followed prospectively for two years with 40 patients in each group. At the one year follow up time frame, patients in the debridement/lavage and HA group were found to have statistically longer lasting improvement in walking pain, night pain, and ability to walk 100 meters with no complications observed. It was concluded that the post-arthroscopic instillation of a HA-based synovial fluid substitute into the joint is a suitable way of achieving long-term stabilization of the treatment outcome. However, their study did not utilize WOMAC and SF-36 questionnaires to evaluate functional efficacy.

Although differences in the patient population and indication for arthroscopy exist when compared to our study, these above published results correlate with our hypothesis that HA delivered at the time of arthroscopy for osteoarthritis of the knee can be performed safely with the preliminary data showing that the combined procedure is efficacious in improving patient's pain level and functional outcome.

Another key factor in determining success of the arthroscopy procedure for knee OA is patient selection. In a recent review of evidence based factors influencing arthroscopy for knee OA by Darling et al [40], the authors recommended arthroscopy for OA only in patients with short duration of symptoms, medial sided knee pain with localized tenderness, preservation of the joint space on radiograph, and mechanical symptoms. Patients with mechanical mal-alignment, flexion contracture, and obesity are not likely to have long term improvement after arthroscopy for knee OA. The above statements were also supported by several other studies [2,11,41]. The results from our study and other literature reports support the notion that the addition of HA injection at the time of the arthroscopy procedure for knee OA will result in better pain relief with sustained benefits in the right patient population.

There are several limitations in our study. One being that it is a case series, level IV evidence study with small sample size of twenty-three patients at the final follow up (6 months). Another significant limitation is that there were no control group and our data was compared to the historical controls found in literature with a short follow-up time of six months. This presents bias in terms of patient population heterogeneity and patient selection across the different studies. Also majority of our patients had menisectomy in addition to the chondroplasty which could result in the improved outcome scores. However, the strength of this study is that all the procedures were performed by a single surgeon (BB) and the results were collected prospectively. To our knowledge, this is also the first case series study evaluating the preliminary efficacy and safety of combined HA (Orthovisc^®^) injection at the time of arthroscopic debridement for OA of the knee utilizing the WOMAC and SF-36 questionnaires. Complications were also documented for this study group and it was shown that injection of 6 ml/90 mg of Orthovisc^® ^was safe at the 6 months follow up.

## Conclusion

These preliminary data suggest benefits as measured by the WOMAC and SF-36 from arthroscopic debridement plus concomitant HA (Orthovisc^®^) delivery in the treatment of knee OA for this patient group. HA injection of 6 ml/90 mg (Orthovisc^®^) was also found to be safe and without complications at the 6 months follow up time. However, the relative contributions of arthroscopic debridement versus intra-op HA injection for the treatment of knee OA should be evaluated in a future randomized, placebo controlled, double blinded trial.

## Competing interests

Funding and HA (Orthovisc^®^) was provided by DePuy-Mitek.

## Authors' contributions

XL, AS, PF, and BB have contributed to the conception/design, data collection/interpretation, and drafting/revising of the manuscript. RM and JB contributed to the collection and analyzing of the data. BB performed the surgical procedures. All authors approved the final manuscript.
